# A CS-based composite scaffold with excellent photothermal effect and its application in full-thickness skin wound healing

**DOI:** 10.1093/rb/rbad028

**Published:** 2023-04-12

**Authors:** Jing Wang, Shijia Fu, Huishan Li, Yue Wu

**Affiliations:** School of Life Sciences, Northwestern Polytechnical University, Xi'an 710072, China; School of Life Sciences, Northwestern Polytechnical University, Xi'an 710072, China; School of Life Sciences, Northwestern Polytechnical University, Xi'an 710072, China; School of Life Sciences, Northwestern Polytechnical University, Xi'an 710072, China

**Keywords:** skin regeneration, chitosan, carbon nanotubes, hydroxyapatite nanoparticles, photothermal effect

## Abstract

The development of natural polymer-based scaffolds with excellent biocompatibility, antibacterial activity, and blood compatibility, able to facilitate full-thickness skin wound healing, remains challenging. In this study, we have developed three chitosan (CS)-based porous scaffolds, including CS, CS/CNT (carbon nanotubes) and CS/CNT/HA (nano-hydroxyapatite, n-HA) using a freeze-drying method. All three scaffolds have a high swelling ratio, excellent antibacterial activity, outstanding cytocompatibility and blood compatibility *in vitro*. The introduction of CNTs exhibited an obvious increase in mechanical properties and exerts excellent photothermal response, which displays excellent healing performance as a wound dressing in mouse full-thickness skin wound model when compared to CS scaffolds. CS/CNT/HA composite scaffolds present the strongest ability to promote full-thickness cutaneous wound closure and skin regeneration, which might be ascribed to the synergistic effect of photothermal response from CNT and excellent bioactivity from n-HA. Overall, the present study indicated that CNT and n-HA can be engineered as effective constituents in wound dressings to facilitate full-thickness skin regeneration.

## Graphical abstract

**Figure:**
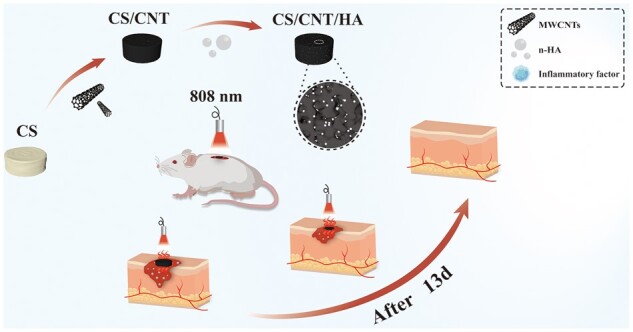

## Introduction

As the largest organ in mammals, the skin plays a crucial role in sensory detection, fluid homeostasis and protection against external damage, thereby contributing to maintaining normal metabolic activity [1–4]. However, the protective functions of the skin are impaired by full-thickness skin defects, arising from aging, burns, surgery, mechanical or chemical trauma, and other superficial injuries, leading to serious wound infections and potentially life-threatening complications [5, 6]. Besides, a severe wound may affect health and even threaten people’s lives, as well as placing a huge financial burden on patients [7, 8]. Therefore, it is essential to design and develop a wound dressing that can promote wound healing, improve skin regeneration and inhibit scar formation [9]. An ideal wound dressing should have desirable biocompatibility, adequate mechanical properties and excellent cellular regulatory ability [1]. Several researchers have investigated that the scaffolds with 3D network porous structures could enhance matrix permeability, promote nutrient diffusion and facilitate cell adhesion and growth [10–12]. Furthermore, the porous scaffold has good water retention, which is conducive to skin tissue repair by keeping the wound area moist [13].

Recently, wound dressings prepared from natural polymers, such as chitosan, sodium alginate, gelatin and hyaluronic acid have attracted extensive attention due to their excellent biocompatibility and hydrophilicity [14–18]. Among these materials, chitosan, the only naturally occurring cationic polysaccharide, has been widely used in biomedical applications [19, 20]. In particular, chitosan has good biocompatibility, biodegradation, antibacterial and wound healing properties, which is a suitable scaffold material for tissue regeneration [21].

Carbon nanotubes (CNTs), first reported in the 1990s, were made up of fibrous structures with a nanosized diameter and micro-sized length [22]. CNTs are materials with ultrahigh surface area and excellent thermal conductivity. The robust mechanical strength and high chemical stability of CNTs have turned them into promising fiber reinforcement materials in tissue regeneration scaffolds [23–25]. It is important to note that CNTs exhibit a stronger ability to promote wound healing than other non-metallic nanostructures [26]. Moreover, multi-walled CNTs (MWCNTs) are more suitable for tissue engineering applications due to their size and can be used as focal adhesion sites to enhance cell adhesion for anchorage-dependent cells such as fibroblasts [27, 28]. It has been reported that CNT had an excellent photothermal effect. As we know, NIR light at 808 nm has excellent penetration into biological tissues, which can reduce inflammation, accelerate tissue metabolism and promote tissue regeneration [29, 30]. The photothermal effect produced by NIR light can cause a rapid increase in local temperature, which can damage bacterial membranes and proteins, and render bacteria inactive [31, 32]. It has been proved that tissue cells can tolerate relatively low temperatures in the range of 50–60°C for a short period, whereas temperatures over 60°C can scald surrounding tissue within a short time. Moreover, the photothermal property under NIR stimulation was beneficial to the antibacterial property of scaffolds. Therefore, CNT was employed in our study to form composite scaffolds with CS, to promote the wound healing effect by taking advantage of its photothermal properties.

Hydroxyapatite (HA) has a similar composition to the inorganic component of natural bone, and it owned excellent biocompatibility and biodegradability [33]. Based above, HA has great potential in histological applications [34]. The chemical structure of nano-HA (n-HA) was very similar to the biomineralized structures and presented excellent biological activity without causing inflammatory reactions [35, 36]. Previous studies focused on n-HA showed a promising prospect in tissue engineering [37–39], hence, it was employed in this study to improve the bioactivity of CS-based composite scaffolds.

Based on the above description, the study herein attempts to develop a series of CS-based antibacterial scaffolds with good biocompatibility and bioactivity, which can effectively promote skin tissue repair. Three kinds of CS-based porous scaffolds, CS, CS/CNT and CS/CNT/HA, were prepared by freeze-drying. Their physicochemical properties including porous structure, degradation properties, swelling behaviors and photothermal response were characterized. Then, the antibacterial properties, and the biocompatibilities *in vitro* and *in vivo* were evaluated, and their capacities to induce skin tissue healing was further investigated in the mouse model. Meanwhile, the role of the photothermal effect in skin wound healing was specially assessed and discussed.

## Materials and methods

### Preparation of scaffolds

#### Chitosan scaffolds

CS solution was prepared by dissolving 2 g of CS into 100 ml of 2% acetic acid solution (Sinopharm, China, diluted by ultrapure water) via stirring at ambient temperature for 30 min. Then, it was sonicated for 20 min to remove the air bubbles. After being poured into a 24-well culture plate, the CS solution was precooled at −20°C for 24 h and then lyophilized for another 48 h to form the porous scaffolds. To remove the residual acetic acid inside the scaffolds, 1% NaOH solution (Sinopharm, China) was used to soak for 10 min. Subsequently, the materials were washed with ultrapure water five times until the soaking solution of scaffolds was close to a physiological pH. Hereafter, the scaffolds were precooled at −20°C for 24 h and lyophilized for 48 h again to obtain the final porous scaffolds.

#### CS/CNT scaffolds

MWCNTs powders were added into the CS solution at a ratio of 2% (m/V) and stirred for 2 h to disperse the MWCNTs uniformly. After then, the mixed solution was sonicated for 30 min to remove the air bubbles and get a more homogeneous solution. The porous scaffolds were prepared as described above.

#### CS/CNT/HA scaffolds

As our previous reports prepared n-HA and dispersed into the mixed CS/CNT solution under stirring. Next, the hybrid solution was sonicated for 20 min to remove the air bubbles. The following steps for scaffold formation are the same as CS scaffold preparation.

### Characterization

#### Scanning electron microscope observation

Scanning electron microscope (SEM) examination was performed to observe the morphology and structure of scaffolds. In detail, after being sputter coated with a layer of gold for 120 s, the samples were viewed by SEM (HITACHI, TM 4000 PLUS).

#### Fourier transform infrared spectra analysis

A Fourier transform infrared spectra (FTIR, IRTracer-100) spectrometer was employed to detect the chemical groups of the scaffolds. For each spectrum, 15 scans were accumulated at 4 cm^−1^ resolution in the range of 4000–400 cm^−1^.

#### X-ray photoelectron spectroscopy analysis

X-ray photoelectron spectroscopy (XPS) was performed on Thermo Scientific ESCALAB 250Xi using a monochromatic Al Kα X-ray source (*E* = 1487.20 eV). The analysis was operated at 14 600 V and 16 mA, and an ultrahigh vacuum pressure of 1.0 × 10^−9^ mBar. A peak of carbon atoms with a single bond (C–C) in C 1 s spectra at 100 eV was used to calibrate the binding energy scale.

#### XRD analysis

X-ray diffractometer (XRD) (Ultima IV) was used to determine the crystalline phase of scaffolds using Cu Kα radiation at 40 kV and 40 mA in the range of 10–60° (2θ). The measurements were limited to the step size of 0.02° and a scan speed of 4° min^−1^.

#### Mechanics performance testing

The mechanical performance of the scaffolds was evaluated using a universal testing machine (INSTRON). In detail for the test of the compressive strength, cylindrical samples (Φ 9 mm × 3 mm) were prepared and three parallel specimens were included in each group. The loading rate was 2 mm min^−1^ and the compressive stress curves were obtained until the samples were compressed to 20% of the original thickness. For the tensile test, samples (22 mm × 7 mm × 2 mm) were prepared and three parallel specimens were included in each group. Before the test, the materials were in a state of swelling and the loading rate was 1 mm min^−1^. The elongation was calculated as the ratio of extensibility to origin length.

#### Swelling behavior investigation

The swelling behavior of the scaffolds was determined by immersing the samples in ultrapure water. Dried samples were weighed (m_0_) before being immersed in ultrapure water. The swollen scaffolds were taken out at regular intervals and weighed again (m_1_) until constant values after removing water from the surface using filter papers. Three parallel specimens were included for each group and the swelling percentage of scaffolds was calculated by the formula *S* (%) = (*m*_1_ − *m*_0_)/*m*_0_ × 100%.

#### Degradation properties

Phosphate buffer saline (PBS; pH 7.4) was used to determine the degradation of scaffolds. Dried scaffolds were weighed (w_0_) and then incubated with PBS at 37°C. After 1, 2, 4, 8 and 30 days, samples were taken out and washed with ultrapure water three times and then frozen again to obtain the weight after degradation, separately. Three parallel specimens were included for each group.

#### Evaluation of the photothermal effects *in vitro*

It is widely acknowledged that MWCNTs can generate heat by light absorption due to their strong NIR absorption [40]. The photothermal properties of CS, CS/CNT and CS/CNT/HA scaffolds at 808 nm were tested in this study. Briefly, the wet samples (CS, CS/CNT or CS/CNT/HA) were irradiated continuously by NIR laser (808 nm, 0.5 W) for 5 min. The temperatures of the samples were recorded every 10 s using a NIR thermal imaging device (ST 8450). Irradiation time–temperature curves were then plotted for evaluating the photothermal ability of different samples. Furthermore, the samples underwent continuous infrared irradiation and natural cooling four times to measure the photothermal stability and the temperature was recorded every 30 s. Three parallel specimens were also included in each measurement.

### Biocompatibility evaluation

#### Cell culture

HUVEC and L929 cells were employed to investigate the biocompatibility of scaffolds. Both the cells were incubated at 37°C, 5% CO_2_ and 95% relative humidity in a cell culture incubator and were sub-cultured at 80% confluence.

#### MTT assay

MTT assay was used to determine the effect of the material on cell proliferation. Three kinds of scaffolds (Φ 5 mm × 1 mm) were sterilized by immersing in 75% alcohol for 30 min and then exposed to UV light for 120 min for both sides. After being placed into 96-well culture plate, HUVECs and L929 cells were seeded, respectively, on them at a density of 5 × 10^4^ cells well^−1^. After culturing for 1, 3 and 5 days, 10 μl of MTT reagent (0.5 mg ml^−1^) was added to each well and incubated for 4 h. Hereafter, the culture medium was removed carefully, and Formazan Solubilization Solution was added for 100 μl each well. The plate was then shaken on a shaker for 10 min to dissolve the formazan crystals. Finally, the absorbance values of the solving liquid were read at 570 nm using a multifunctional microplate reader (Bio-Tek, USA).

#### SEM observation

After the sterilized samples were placed into a 24-well culture plate, L929 cells were seeded on the surface, respectively. After 3 days, the medium was removed and the cells on scaffolds were fixed with 4% paraformaldehyde overnight. Subsequently, the cells were washed two times using PBS and dehydrated in gradient ethanol (30%, 50%, 70%, 90% and 100%) (v/v) for 20 min each step and dried at ambient temperature. After that, the scaffolds were sputter-coated with a layer of gold for SEM viewing.

#### Phalloidine staining

Leaching solutions were used to investigate the effect of scaffolds on cell’s cytoskeleton arrangement. For leaching solutions preparation, the three sterilized materials were soaked in complete growth media (DMEM containing 10% fetal bovine serum and 1% Penicillin/Streptomycin) at 37°C sterile incubator for 48 h, respectively. After then, the supernatant was collected as a leaching solution and stored at 4 °C for subsequent cell culture. After L929 cells were seeded in a 24-well culture plate, the leaching solutions were added as a culture medium. After 3 days of culture, cells were washed 2 times with PBS at ambient temperature and then fixed in 4% paraformaldehyde for 40 min. Subsequently, cells were stained with TRITC-conjugated phalloidin (1:200, sigma) in dark for 40 min. After being washed gently, cells were stained with DAPI (1:200, sigma) for 5 min and washed with PBS again. Finally, the cell’s cytoskeleton was visualized by confocal laser scanning microscopy (Leicasp5, Germany) and fluorescence microscope (80i, Japan).

### *In vitro* antibacterial performance

The bacteria Gram-positive *Staphylococcus aureus* and Gram-negative *Escherichia coli* were used to evaluate the antibacterial performance of materials. The bacteria suspension with an optical density (OD, 600) was prepared and then co-cultured with three kinds of sterile scaffolds (37°C, 120 rpm) for 24 h. After then, 100 μl of each bacterial solution was taken out and diluted with 500 μl of sterile water. The bacterial density was recorded by a UV spectrophotometer microplate reader to detect the OD value at 600 nm, and the one without material served as a control. The bacteriostasis rate was calculated as % = (OD_control_ − OD_sample_)/OD_control_ × 100%. Three parallel experiments were included for each sample. At the same time, the materials in each group were fixed with 4% paraformaldehyde and the morphology of bacteria on the material surface was observed by SEM. For the plate-counting assay, 100 μl of bacterial solution in each group was taken out and plated onto the agar plate and cultured for 48 h to observe and record the formation of bacterial colonies. The bacteriostatic ring assay was also employed to evaluate the antimicrobial activity of different scaffolds. After 100 μl bacterial solution with an OD was plated onto the agar plate, the sterile specimens (Φ9 mm × 1 mm) were placed at a certain distance. After incubating for 24 h at 37°C, the inhibition zone size was captured and measured with vernier calipers.

### *In vivo* wound healing

All animal protocols were approved by the Institutional Animal Care and Use Committee of Northwestern Polytechnical University. BALB/C (6 weeks old, 20 g, male) mice were selected to establish the skin defect model.

#### Surgery procedure

Before surgery, all mice were anesthetized by injecting 5% chloral hydrate (0.05 ml g^−1^) with a standard procedure, and then the back hair was shaved and disinfected with iodophor. A skin wound (Φ = 6 mm) was created on the back and sterile scaffolds (Φ = 6 mm) of CS, CS/CNT and CS/CNT/HA were used to cover the wounds. For each scaffold, a conventional group and a photothermal group were designed for subsequent treatment, and the wound without any treatment was set as the control group. For photothermal treatment, the wound was exposed to NIR (808 nm, 0.5 W) for 20 s each time, and repeated twice every day.

To examine the histocompatibility of three kinds of scaffolds *in vivo*, an airbag model was also established. In detail, after the mice were anesthetized and shaved, a cut was made on the skin and then separated bluntly to produce an air bag. The mice were randomly divided into three groups and the sterile scaffolds of CS, CS/CNT and CS/CNT/HA were implanted, respectively.

#### Wound treatment and result analysis

As been reported widely, the photothermal properties of the material *in vivo* were investigated by exposing the scaffolds that were implanted into the skin to 808 nm near-infrared (NIR) light for 20 s each time and twice a day. NIR light at 808 nm has excellent penetration into biological tissue. Besides, NIR light-mediated photothermal therapy belongs to a physical method which will not produce little side effects on the body [41]. In this study, the wounds of different groups were photographed on Days 0, 3, 7 and 14. The relative area of wounds was calculated according as follows: relative wound area (%) = *A*_t_/*A*_0_ × 100%, where *A*_0_ is the wound area at Day 0 and *A*_t_ is the wound area at Day *n* (*n* = 0, 3, 7 and 13). Three mice in each group and each time point were used to count. After 13 days, the mice were sacrificed and the skin with wound sites was collected. Meanwhile, the scaffolds in the airbag were also collected on Days 3, 7 and 14, respectively. All samples were fixed in 4% paraformaldehyde, embedded in paraffin, and then sliced for subsequent histological analysis. Hematoxylin and eosin (H&E) staining was employed to visualize the degree of skin repair and cell distribution at the interface between tissue and scaffolds. Masson Trichrome (Masson) and Sirius red staining were also employed to determine the newly formed collagen at the wound sites. All samples were photographed and analyzed using a fluorescence microscope (80i, Japan). The proportion of collagen deposition in the wound area was measured by Image-Pro Plus 6.0.

#### Blood compatibility

The blood compatibility was performed by adsorbing plasma and whole blood on scaffolds [41]. Blood was collected from BALB/C mice using a 1.5 ml centrifuge tube. Platelet-rich plasma was separated from the blood by centrifuging at 4000 rpm for 5 min. Plasma and whole blood were added to the surface of the materials, which were fixed overnight with 4% paraformaldehyde. After dehydration with alcohol, the scaffolds were allowed to dry at ambient temperature. Finally, the SEM images were obtained.

### Statistical analysis

Statistical analysis was carried out using GraphPad Prism 8 to analyze the differences between the groups. The data were presented as means ± standard deviation (SD). Differences between paired data were compared using the *t*-test and two-way ANOVA for multiple comparisons. Compared to the control group, a significant difference was defined as ****P < 0.0001.

## Results and discussions

### Morphology and structure of scaffolds

**Figure 1.**
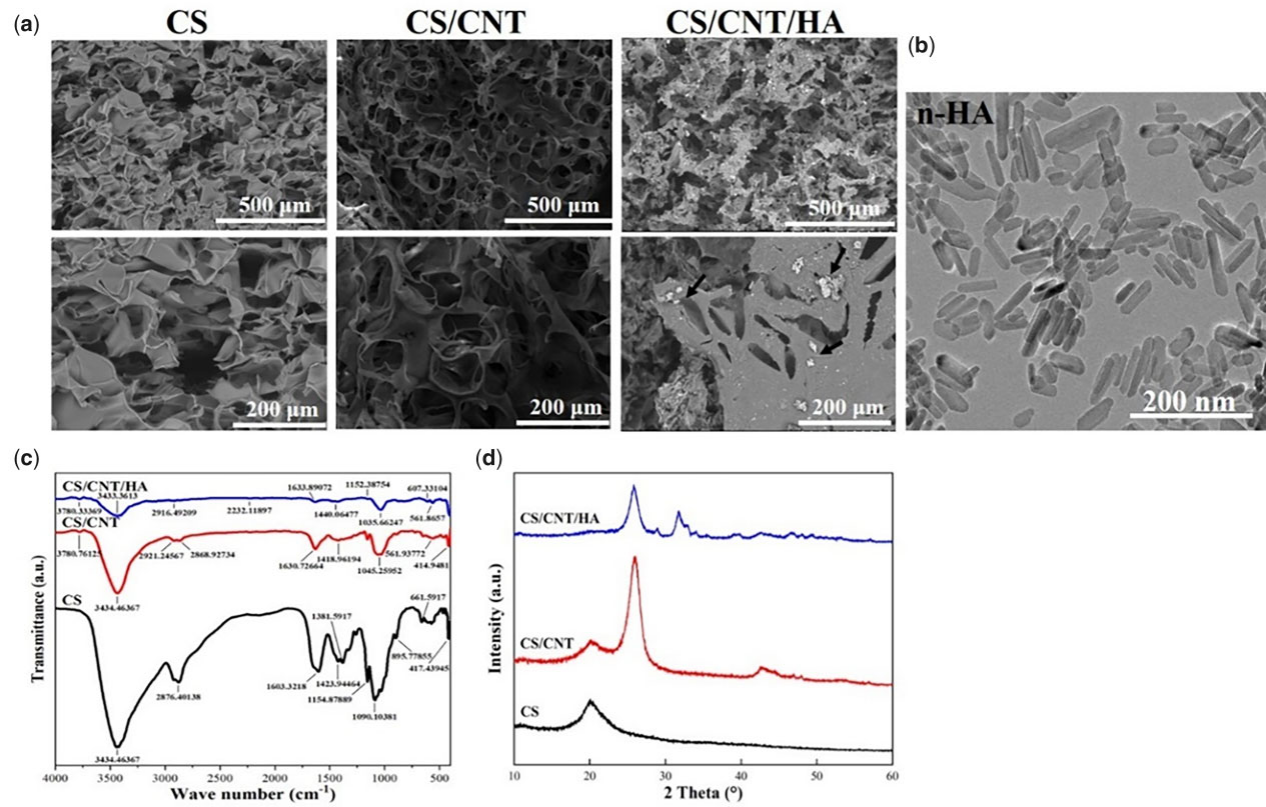
SEM images of CS, CS/CNT and CS/CNT/HA scaffolds (black arrow indicated the n-HA in scaffolds) at low and high resolution (**a**); TEM image of n-HA particles (**b**); FTIR spectra of CS, CS/CNT and CS/CNT/HA scaffolds (**c**); XRD patterns of CS, CS/CNT and CS/CNT/HA scaffolds (**d**).

The morphologies of the obtained three kinds of CS-based scaffolds were shown in Fig. 1a. All three scaffolds exhibited a 3D network structure and were full of interconnected pores, and the macropore size (∼100–200 µm) and distribution were similar in the three groups. The addition of CNTs and n-HA particles did not change the structure of the scaffolds and the n-HA particles were distributed evenly in the pores and surface of the scaffolds in micron size as indicated by the black arrow. As shown in Fig. 1b, the TEM image of n-HA particles before being added into scaffolds exhibited a nanorod morphology with a mean length of 76.2 ± 8.326 nm and width of 19.6 ± 1.173 nm. It indicated an agglomeration occurred after the n-HA particles were introduced into the scaffolds.

The comparisons of FTIR spectroscopic analysis of CS, CS/CNT and CS/CNT/HA were exhibited in Fig. 1c. The peaks located at 3434 cm^−1^ were assigned to N–H and O–H stretching vibration, and the bands at 2876 cm^−1^ correspond to the C–H stretching. The stretching and bending vibrations of ${\text{PO}}_{4}^{3-}$ occurred at 1035 and 561 cm^−1^, respectively [42].

By comparison, the spectrum in CS/CNT and CS/CNT/HA composite scaffold at 3434 and 1035 cm^−1^ were weakened than CS, which assigned to the N–H stretching vibration and N–H bending vibration in the primary amine group. The attenuated bands in CS/CNT and CS/CNT/HA composite scaffold at 1630 cm^−1^ was the N–H bending vibration of deformation amino groups in the amide group [43]. All these might be ascribed to the interaction between –COOH in the carboxylated CNTs and –NH_2_ in CS that will be leading to a reduction. The FTIR results indicated there were mainly physical interactions rather than chemical reactions between CS and CNTs in the composite scaffolds.

Figure 1d exhibits the XRD results of the three scaffolds. For CS scaffolds, only a broad diffraction peak at 20.0° was found, indicating that the CS in the sample was in an amorphous state [43]. This result may be caused by freeze-drying. Similar phenomena were also reported in other research [44]. The main characteristic peaks of CS/CNT were observed at 20.0° and 26.0°, respectively. The addition of CNTs still maintained the characteristic peaks of CS, further indicating a physical interaction rather than a chemical reaction between CS and CNTs. The XRD pattern of the CS/CNT/HA scaffold showed three intense peaks of crystal phases at 25.9°, 32.0° and 39.7° (2θ) were, respectively, assigned to (002), (211) and (310) of HA. The broad peaks with poor crystallinity around the characteristic diffraction region near 32° (2θ) suggested a low crystallinity of n-HA in the composite scaffold, which was similar in crystallinity to natural bone minerals [45]. It also pronounced that n-HA particles have existed in CS/CNT/HA composite scaffold. XPS analysis was also used to evaluate the surface elements and element proportions of three kinds of scaffolds.

**Figure 2.**
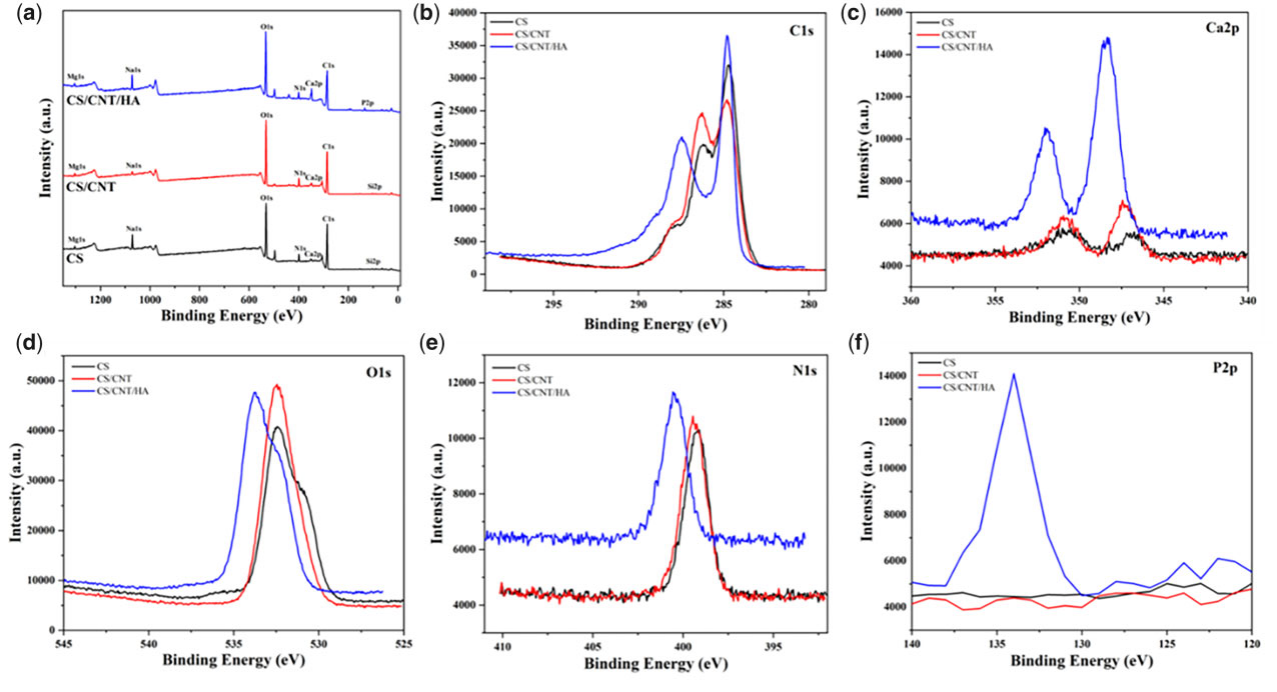
Full spectrum of XPS survey scan for CS, CS/CNT and CS/CNT/HA scaffolds (**a**); high-resolution XPS spectra of signal C1s (**b**), Ca2p (**c**), O1s (**d**), N1s (**e**) and P2p (**f**) element for CS, CS/CNT and CS/CNT/HA scaffolds.

As shown in Fig. 2, the XPS survey scans for CS, CS/CNT and CS/CNT/HA showed characteristic binding energy peaks at 285, 398 and 533 eV due to the presence of C, N and O elements [46]. The addition of CNTs did not change the characteristic peaks of CS, which again proved that CS and CNTs physically interacting. The results are consistent with those of FTIR and XRD. Compared with CS and CS/CNT scaffolds, the peak value of Ca2p in CS/CNT/HA was increased obviously. Furthermore, CS/CNT/HA presented an additional peak at binding energy 130 eV, which was a characteristic peak of P2p. These results also indicated the presence of n-HA in the CS/CNT/HA composite scaffold.

### Mechanics performance of scaffolds

**Figure 3.**
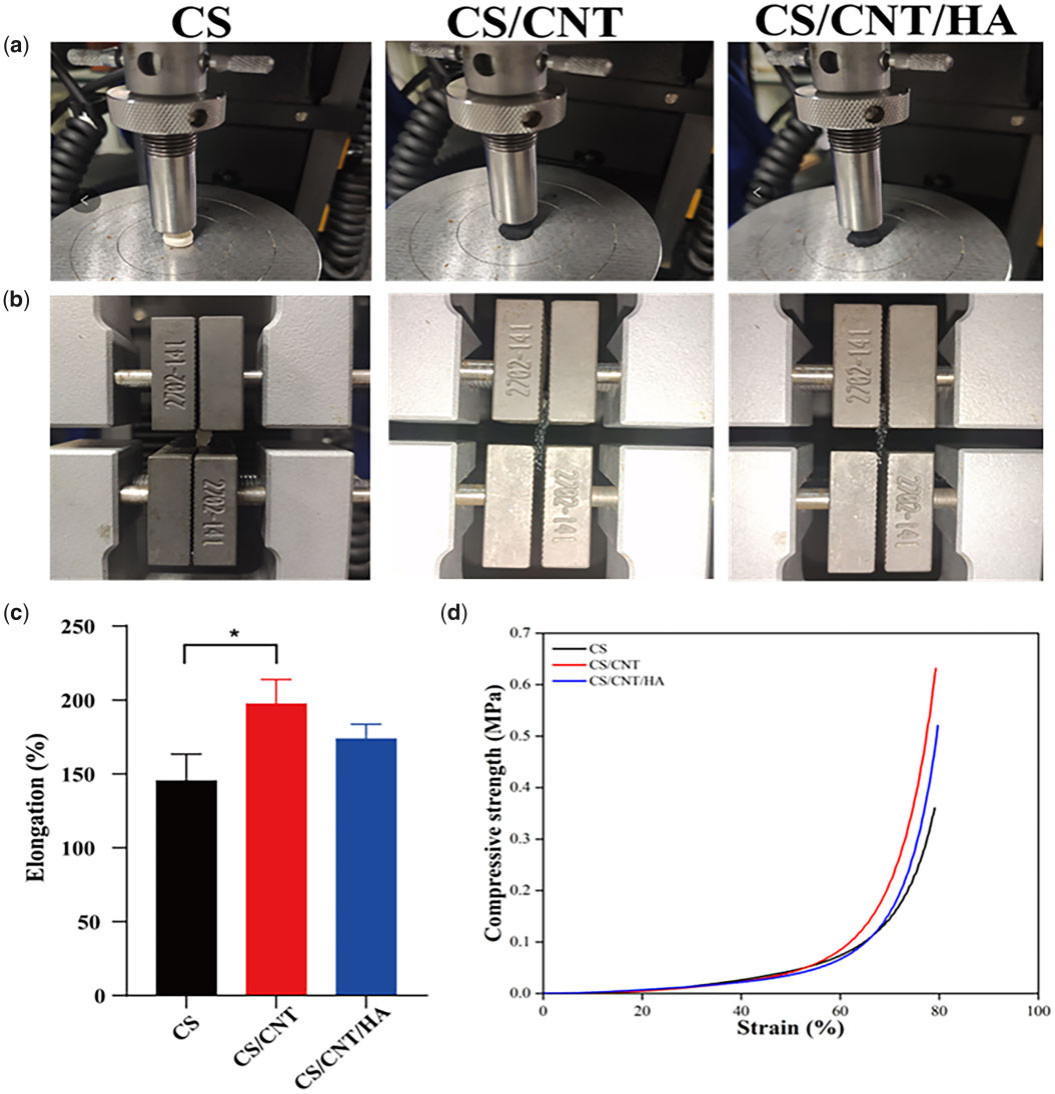
The schematic diagram and results of the compressive test (**a** and **c**) and extension test (**b** and **d**). *P < 0.05.

Mechanical property is an important property which potentially used in tissue engineering. An excellent mechanical strength can avoid undesired deformation of biomaterials due to external forces such as extrusion during use [47]. As others reported, the outstanding strength and elasticity of CNTs can provide excellent reinforcement in the composite scaffold, which can be strongly combined with the matrix and improve the mechanical strength of the materials [48]. As shown in Fig. 3c, the compressive strength of the CS scaffold was only 0.3604 MPa, but it was increased to 0.63197 and 0.5207 MPa after the addition of CNTs and n-HA, respectively. By calculation, it was enhanced by 75.3% for CS/CNTs and 44.4% for CS/CNTs/HA. The elongation of three scaffold materials was evaluated and the results were presented in Fig. 3d. It was evident that the introduction of CNTs significantly improved the flexibility of the CS-based scaffolds. The average elongation value could reach 197.4% and 173.6% for CS/CNTs and CS/CNTs/HA, respectively, while the CS scaffold was only 145.3%. It was worth noting that the mechanical properties of the composite scaffolds tend to decrease after the introduction of n-HA, this might be ascribed to the n-HA particles being easily agglomerated and unevenly distributed in the matrix, thus reducing the interface binding between the particles and the matrix [49]. In any case, these two composites greatly improve the mechanical properties than that of CS scaffolds.

### Swelling properties of scaffolds

**Figure 4.**
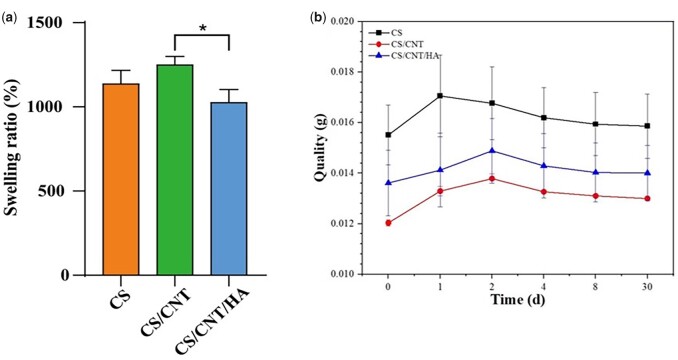
The swelling properties (**a**) and the degradation behaviors (**b**) of CS, CS/CNT and CS/CNT/HA scaffolds. *P < 0.05.

Swelling performance is an important parameter of the 3D scaffolds in biomedical applications such as tissue engineering or wound healing. The swelling properties of scaffolds are essential for the absorption of body fluids and the transfer of cellular nutrients and metabolites [50]. A higher swelling rate is more conducive to absorbing wound exudate and promoting wound healing [51]. Figure 4a shows the swelling performance of the scaffolds in ultrapure water. The CS/CNT scaffold showed the most distinct swelling behaviors (1249%), and 1137% and 1026% for CS and CS/CNT/HA, respectively. In the process of swelling, CS scaffold exhibited a shorter equilibrium swelling time, ascribing to the –COOH in the carboxylated CNTs could reduce the hydrophobicity of the CNTs. The CS/CNT/HA showed the lowest swelling rate, which is ascribed to the addition of n-HA. The swelling capacity of wound dressing represents the level of water absorption capacity and is an important factor to evaluate biological materials. It has an important role in the antibacterial activity and the wound dressing can absorb a small amount of the wound exudate by swelling, which helps quick restoration of the wound and decreases the risk of infection. The swelling properties of a wound dressing depend on the nature of the scaffolds and the swelling environment, and each variety in the structure of scaffolds itself affects its swelling behavior [52]. In this study, the filling of pore spaces by n-HA particles inside the scaffolds can stop the water molecules from entering the scaffold. Although the swelling ratio in CS/CNT/HA was slightly lower than in the other groups, it did not seem to make a negative impact on the eventual repair effect.

### Degradation properties of scaffolds

The biodegradability of the scaffolds is another important indicator that needs to be considered in tissue engineering applications [53]. Degradation performance of three kinds of scaffolds was conducted in PBS (pH ≈ 7.4), and all three scaffolds exhibit almost no degradation in the first 3 days (Fig. 4b). It might be caused by the swelling effect of the scaffolds or the hydrophobicity of CNTs [54]. After degradation for 4 days, the quality of the samples in each group showed a slight decrease. The stability of scaffolds could maintain the integrity of the structure *in vivo* during the repair process which was advantageous for wound healing. However, it does not seem to be consistent with the results *in vivo* as shown in H&E staining images (Fig. 9). *In vivo*, the Lysozyme could catalyze the hydrolysis of the 1,4-β bond between n-acetylenic acid and N- to promote the degradation of CS. When the composite scaffold is implanted in the body, water penetrates the scaffold to expand, and lysozyme reacts with the CS active site in the pore wall of the scaffold. As the reaction develops, the CS macromolecules are cut by lysozyme, and more CS macromolecules are exposed to the lysozyme, leading to the thinning of the scaffold wall and the collapse and destruction of the scaffold, and finally exhibits a faster degradation rate *in vivo* [55, 56]. In general, the degradation rate in physiological condition matched the rate of skin tissue regeneration well.

### *In vitro* photothermal performance of scaffolds

**Figure 5.**
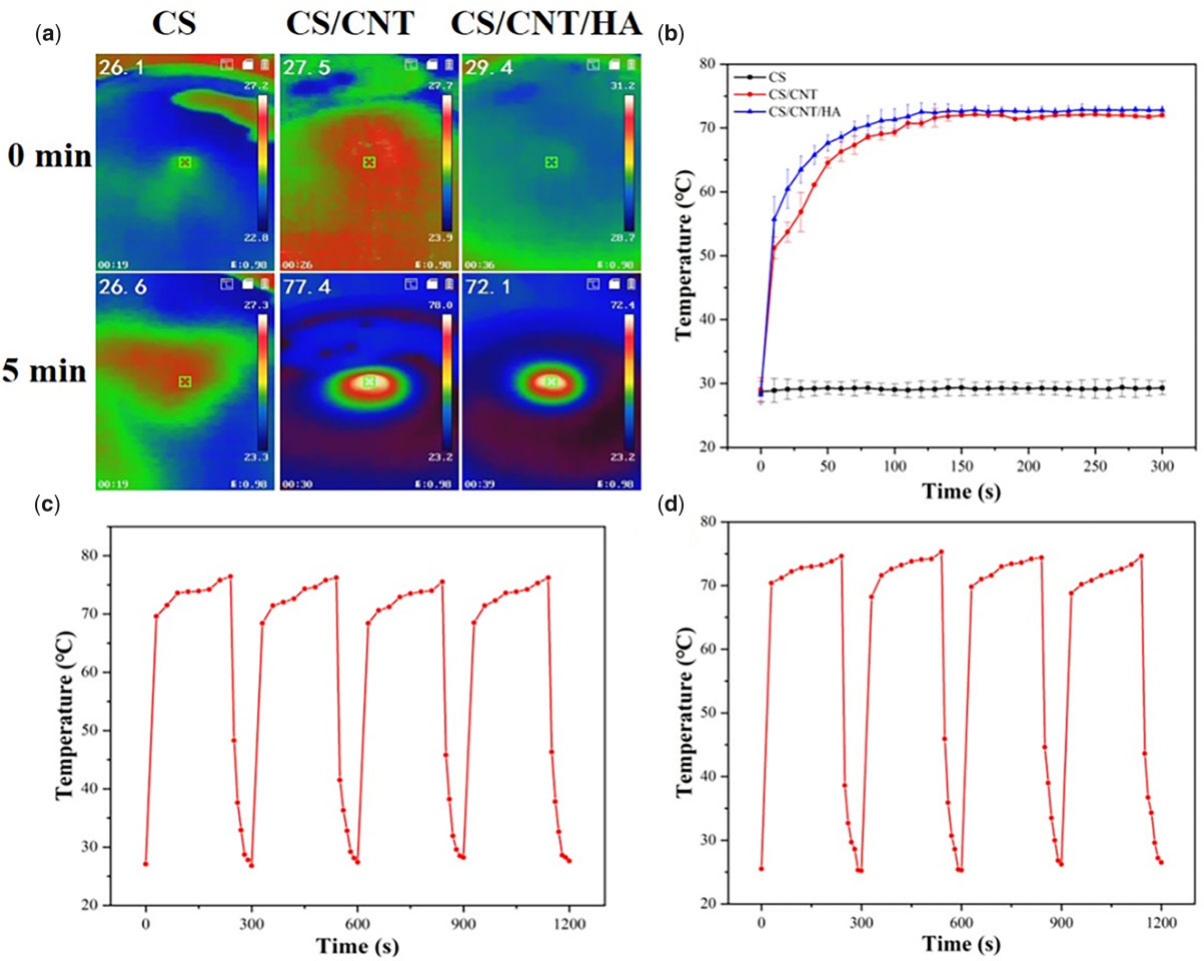
The photothermal properties of CS, CS/CNT and CS/CNT/HA scaffolds. Photothermal images (**a**); time–temperature curve of CS, CS/CNT and CS/CNT/HA scaffolds exposed to 808 nm NIR irradiation at a 1.0 W cm^−2^ output power intensity for different times (**b**); the heating and cooling cycle curve of CS/CNT (**c**) and CS/CNT/HA (**d**) scaffolds exposed to 808 nm NIR irradiation at a 1.0 W cm^−2^ output power intensity for four cycles.

The photothermal behaviors of three scaffolds were examined via exposure to 808 nm NIR. The results (Fig. 5) showed that NIR laser irradiation at 808 nm increased the temperature of the CS/CNT and CS/CNT/HA composite scaffolds instantly. CS/CNT scaffold had a rapid temperature rise from 28°C to 72°C under laser irradiation for 240 s and the temperature of the CS/CNT/HA scaffold reached 73°C. It pronounced that both CS/CNT and CS/CNT/HA scaffolds had an effective absorption of NIR light. However, the CS scaffold had no obvious temperature rise in the same conditions. Moreover, CS/CNT and CS/CNT/HA composite scaffolds still maintained extraordinary photothermal stability even after four cycles of on–off NIR irradiation as the maximum temperature of the elevation had a slight change, indicating that the materials had good photothermal stability. It is promising for them in the fields of antibacterial and photothermal therapy in tissue engineering.

### Effects of different scaffolds on cell behavior

Morphological changes of cells that were exposed to biomaterials or cultured in the leaching solution of materials indicated their toxicity [45]. Many studies have shown that a material with a highly porous structure would provide more space for cells to attach and grow, and thus have better biological properties [56].

**Figure 6.**
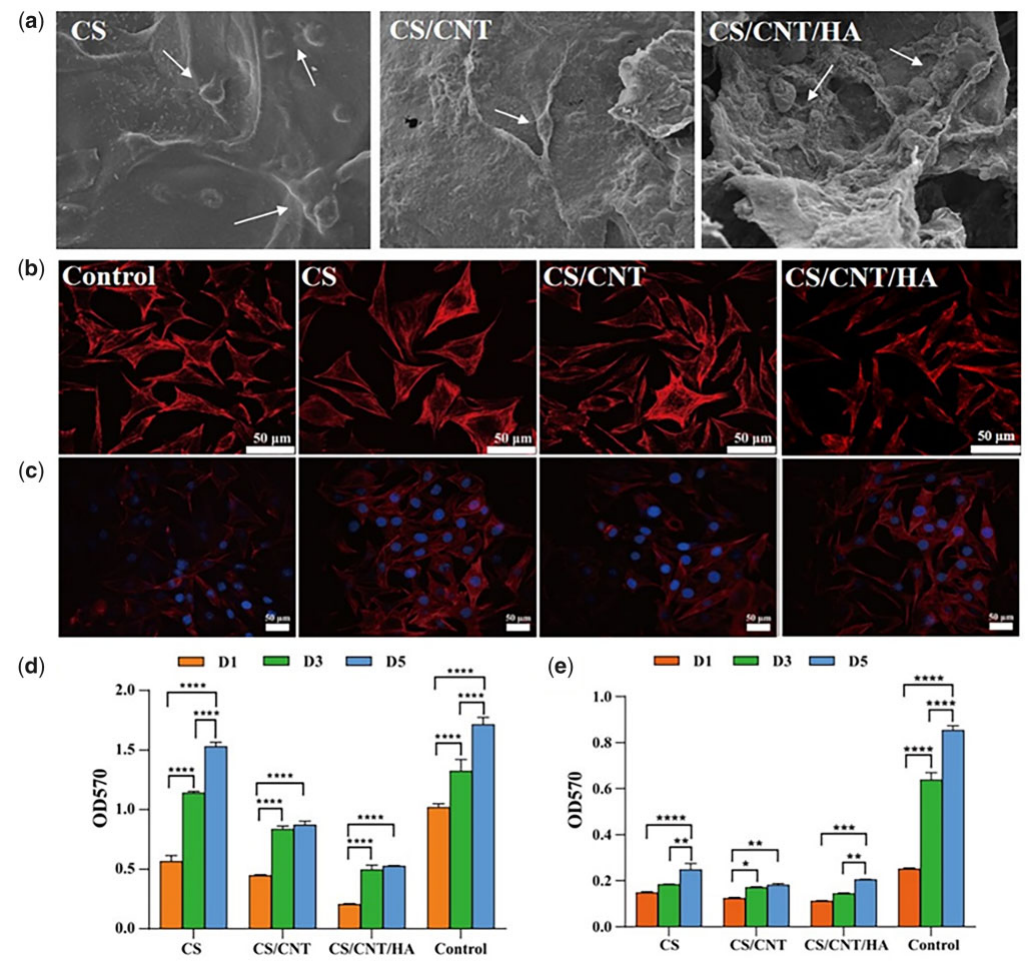
The biocompatibility of CS-based scaffolds. SEM images of L929 morphology on CS, CS/CNT and CS/CNT/HA scaffolds after incubation for 2 days (**a**); the cell cytoskeleton and nucleus images of L929 co-cultured with control and the extracts from CS, CS/CNT and CS/CNT/HA scaffolds (**b** and **c**); proliferation results of the L929 (**d**) and HUVECs (**e**) co-cultured with control, CS, CS/CNT, CS/CNT/HA scaffolds, respectively. *P < 0.05, **P < 0.01, ***P < 0.001 and ****P < 0.0001.

Figure 6a shows the spreading morphology of L929 cells growing on the surface of three scaffolds after 3 days, respectively. The cells on all the surfaces of CS, CS/CNT and CS/CNT/HA composite scaffolds were spreading well and showed no obvious difference. The cells were spindle-shaped and polygonal, and the cell filopodia were connected. At the same time, the cells on the surface of all three scaffolds were evenly distributed. These results indicated that L929 cells were well attached to the scaffold surface, and all three scaffold materials did not impact cell viability and were non-cytotoxicity, which also provided a good surrounding environment for cell adhesion and growth. Besides, the cytoskeleton is a crucial component of the cell, which connects to lipid molecules on the cell and nuclear membranes to maintain cell morphology and participate in cell division and motility [57]. We also used the leach liquor of three kinds of scaffolds to inspect the effect of chemical composition on L929 activity and cytoskeleton arrangement. As shown in Fig. 6b, there were obvious pseudopodia and microfilaments in all groups and the cytoskeleton arrangement in the three scaffolds is not different from the control. Moreover, cells formed extensive cell–cell interactions from F-actin/DAPI staining images (Fig. 6c) after 3 days of culture. These findings suggested excellent biocompatibility of CS-based scaffolds for cells. Cell proliferation on the three scaffolds was quantified by performing an MTT assay. Figure 6d and e presents the proliferation of both L929 and HUVECs on CS, CS/CNT and CS/CNT/HA, after 1, 3 and 5 days of culture. It showed a remarkable proliferation by prolonging the culture time on all scaffolds. Combined with the result of SEM observation and cytoskeleton arrangement, it revealed that all three kinds of CS-based scaffolds had the well cellular biocompatibility, which could well promote the attachment, proliferation, and spreading of both HUVECs and L929.

### The potential of the scaffold as a hemostatic material

**Figure 7.**
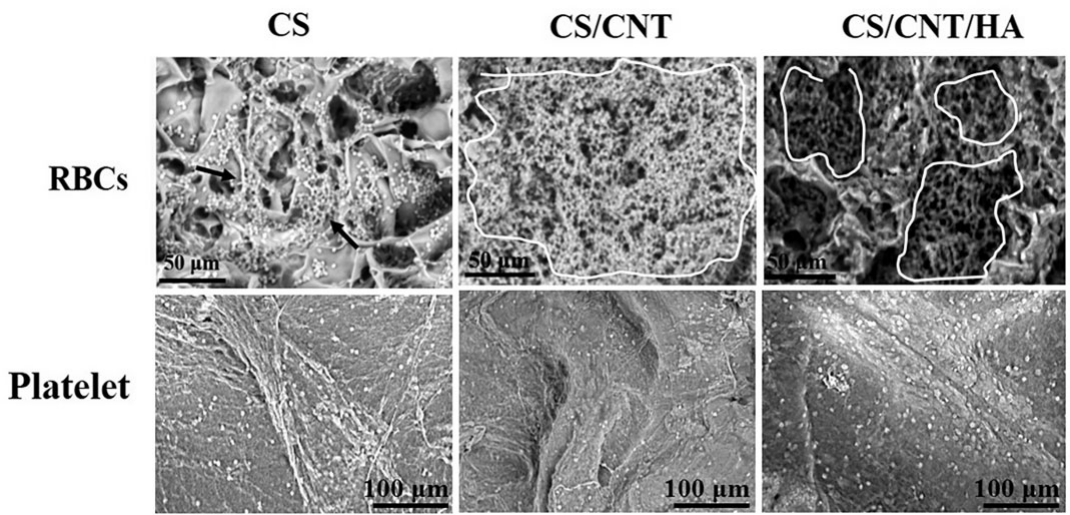
SEM images of blood cells(black arrow in CS group; white circle region in CS/CNT and CS/CNT/HA groups) and platelet adsorption on CS, CS/CNT and CS/CNT/HA scaffolds.

The blood clotting ability is an essential characteristic of hemostatic materials [41]. In Fig. 7, the SEM images show the adsorption of RBCs and platelets on the three scaffolds. In the surface and pores of the composite scaffolds, especially those containing CNTs, a large number of RBCs aggregated (white circle region in Fig. 7a) and widespread platelet pseudopods were observed. The porous structure and electrostatic interaction of the composite scaffolds provide more active sites for the adhesion of RBCs and platelets [58]. The amino groups of CS were protonated to ${\text{NH}}_{3}^{+}$ in contact with the blood, improving the electrostatic interactions with negatively charged RBCs to induce aggregation and adhesion [59]. The existence of MWCNTs increased the surface roughness of the substrate, and it enhanced the high adsorption of RBCs and platelets [60]. Moreover, the platelet on all three scaffolds showed a round shape which indicated an inactive state. As been reported by others, the activated platelet would exhibit an amount of filopodiaper and then spread. Therefore, all three scaffolds could quickly adhere to large amounts of blood, which is conducive to wound coagulation and has the potential for rapid hemostasis.

### Antibacterial properties of different scaffolds

**Figure 8.**
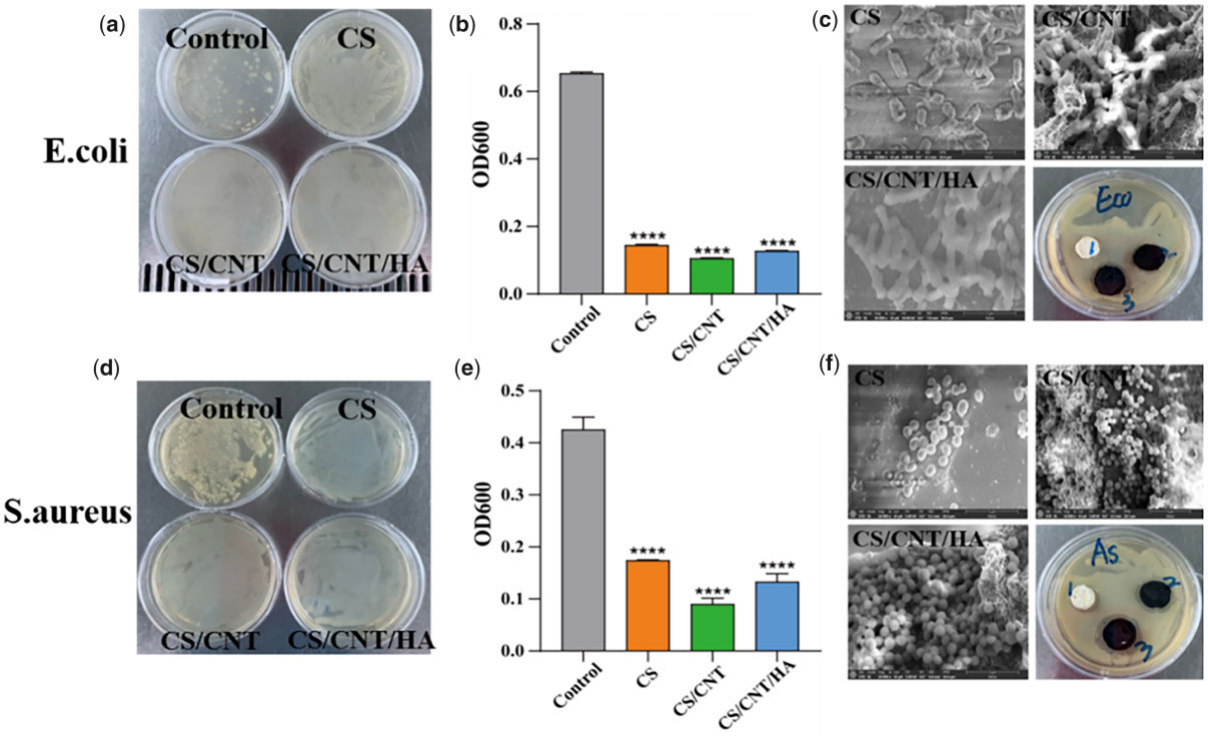
Antibacterial properties of CS, CS/CNT and CS/CNT/HA scaffolds. Images showing the growth status of *E.coli* (**a**) and *S.aureus* (**d**) after co-incubated with control, CS, CS/CNT and CS/CNT/HA scaffolds, respectively. The OD value at 600 nm of *E.coli* (**b**) and *S.aureus* (**e**) after co-incubated with control, CS, CS/CNT and CS/CNT/HA scaffolds, respectively. The SEM images and bacteriostasis circle of *E.coli* (**c**) and *S.aureus* (**f**) after contact with control, CS, CS/CNT and CS/CNT/HA scaffolds. ****P < 0.0001.

The antibacterial activity of materials is crucial for skin tissue engineering since bacterial infections can prevent wound repair by accelerating the formation of exudates during wound healing. The antimicrobial scaffolds can promote the healing process by reducing the inflammatory response at the wound site [61, 62]. As shown in Fig. 8 of our result, the bacterial SEM images (Fig. 8c and f), a large number of bacteria were adsorbed on the surface of the scaffolds, particularly CS/CNT scaffolds, which might be ascribed to their high water absorbency. Moreover, the *E.coli* on the CS surface showed a shrinkage when compared with the other two groups, and *S.aureus* (Fig. 8f) on CS showed a dispersed adhesion state. The difference in morphology between them might be due to different antibacterial mechanisms. Besides, a visible bacteriostasis zone has appeared in all three scaffolds groups. A relative quantitative statistic of antibacterial activity in three scaffolds was also detected by measuring the absorbance of OD 600 with an ultraviolet spectrophotometer. When compared with control (without materials treatment), both the *E.coli* and *S.aureus* suspensions co-cultured with all three CS-based scaffolds showed effective inhibition of the colony formation (Fig. 8b and e), further demonstrating excellent antibacterial activities of materials.

### Biocompatibility *in vivo*

**Figure 9.**
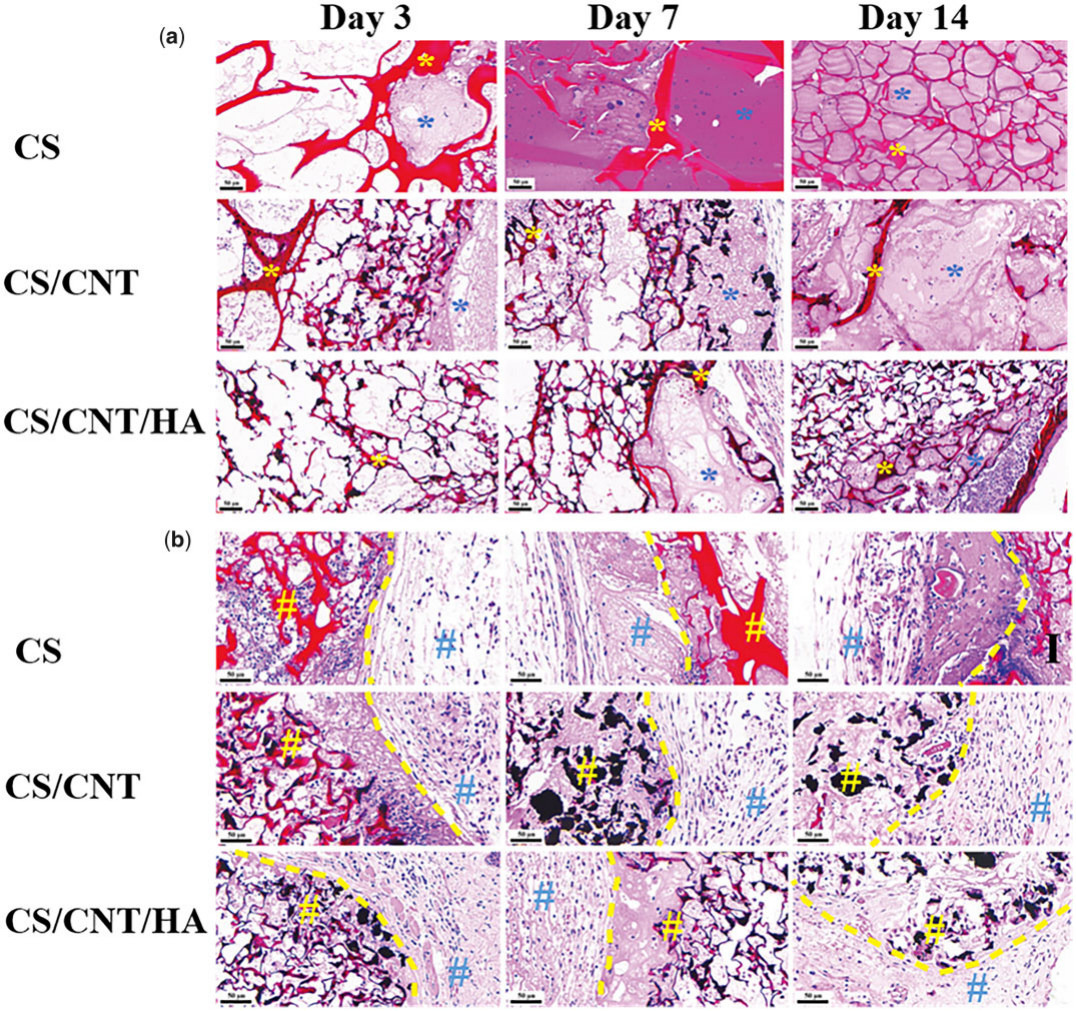
Biocompatibility of CS, CS/CNT and CS/CNT/HA scaffolds *in vivo* after implantation for 3, 7 and 14 days. Histological analysis of tissue growth into the scaffolds and the bonding state (H&E staining, yellow * = scaffolds, blue * = tissue growth into the pores of scaffolds, yellow # = scaffolds site, blue # = host tissue site, yellow dotted line = the interface between host tissue and scaffolds).

As implanted scaffolds, the biocompatibility in the host was essential to evaluate the application prospect of them. In our study, an airbag model was employed to confirm the excellent biocompatibility of CS-based scaffolds during 13 days period. As shown in Fig. 9a, the scaffolds showed an obvious degradation (labeled by a yellow star) with the extension of implantation time, and more and more tissue (labeled by a blue star) growth into the pores. In CS/CNT and CS/CNT/HA groups, the CNT was observed around the implant sites and did not induce obvious inflammation, which is characterized by the excellent integration between scaffolds and host tissue as shown in Fig. 9b. In detail, there was no cystic fibrosis found during the whole investigation period, as well as no amount of inflammatory cell aggregation the interface between host tissue (labeled by blue number sign) and implanted scaffolds (labeled by yellow number sign), and exhibited a tight connection between them.

### Wound healing performance

**Figure 10.**
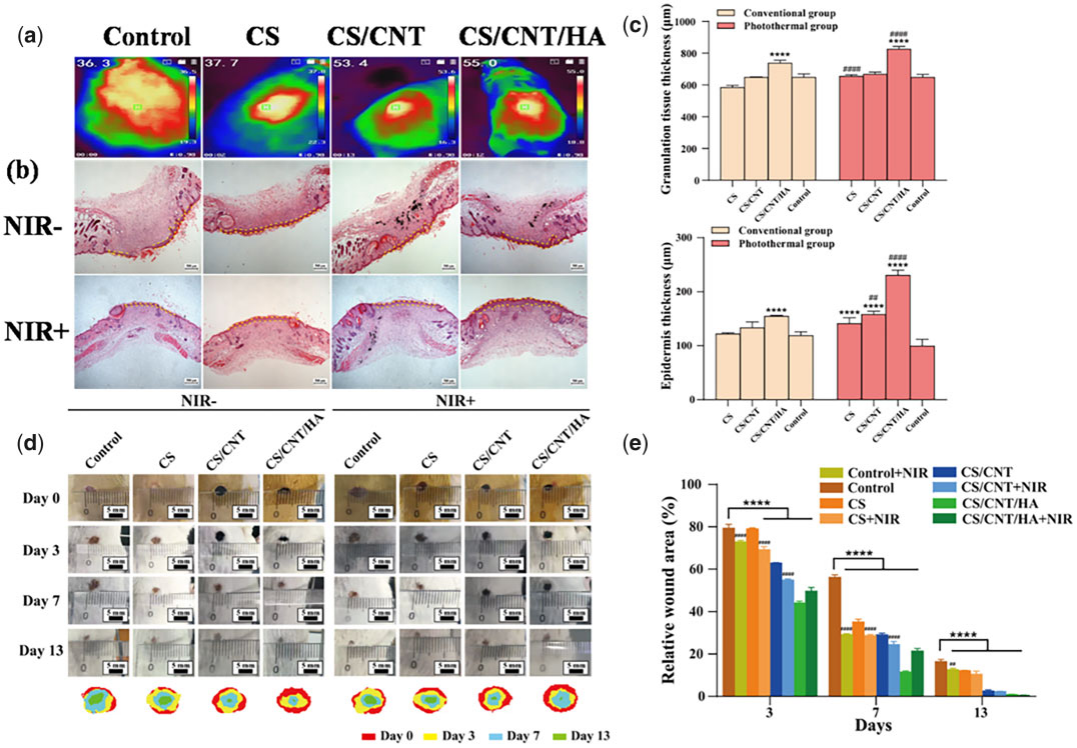
Wound healing properties of CS, CS/CNT and CS/CNT/HA scaffolds *in vivo*. Photothermal images of a mouse wound treated with or without scaffolds and exposed to 808 nm NIR light at 1.0 W cm^−2^ output power (**a**). H&E staining images of wound skin after 13 days of treatment with or without NIR radiation on 808 nm at 1.0 W cm^−2^ output power (**b**). The quantification analysis of the thickness for newly formed granulation tissue and epidermis at the wound sites (**c**). The images of the wound healing process (0, 3, 7 and 14 days) treated by different scaffolds with or without NIR radiation on 808 nm at 1.0 W cm^−2^ output power (**d**). Quantification analysis of wound area at Day 3, 7 and 14 in different groups (**e**) (*difference of scaffolds compared with control, ****P < 0.0001; ^#^differences between the same scaffold with or without NIR radiation on 808 nm at 1.0 W cm^−2^ output power, ^####^*P* < 0.0001).

Three kinds of scaffolds (CS, CS/CNT, CS/CNT/HA) and control (wound without any treatment) were used to investigate the potential in wound healing, and the NIR irradiation groups (CS + NIR, CS/CNT + NIR, CS/CNT/HA + NIR and Control + NIR) were also designed to reveal the role of photothermal effect of scaffolds in wound healing. As shown in Fig. 10a, after irradiation of the wound site with NIR light (808 nm) for the 20 s, the local temperature focused on the scaffolds of both CS/CNT and CS/CNT/HA increased significantly, reaching 53.4°C and 55°C, respectively. The temperature in CS group and control had not increased after NIR irradiation. Compared with the photothermal effect *in vitro* (Fig. 5), the scaffolds of CS/CNT and CS/CNT/HA also had an effective absorption of NIR light *in vivo*.

As the most intuitive evaluation indicator, wound area was used to evaluate the repair effects in different groups [63]. Figure 10d shows representative photographs taken on different days during the wound healing process, directly exhibiting the healing effect of different materials on the wound. Moreover, a schematic diagram and corresponding quantitative analysis of the wound area over time were also shown. Compared with the control group, the other groups presented a significantly enhanced wound repair rate over the whole healing process (*P* ≤ 0.0001), especially under the photothermal condition, indicating that the CS-based scaffolds had an intrinsic promoting effect on wound healing and NIR irradiation also make great contributions to wound healing. H&E staining was employed to determine the histological morphology of skin repair location (Fig. 10b). It was observed that all three scaffolds groups had repaired the defected skin after 13 days in either NIR irradiation or not. However, there were still unrepaired defects in the control group. Besides, the wounds implanted with CS-based scaffolds had completed epithelialization, and the new epidermal hyperplasia was formed obviously. To be more precise, the epidermis thickness was measured to compare the repair effect. As shown in Fig. 10, for the conventional treatment, control showed the smallest epidermis thickness (118 μm), while that in CS, CS/CNT and CS/CNT/HA were 121, 133 and 154 μm, respectively. It showed an obvious pattern of the scaffolds’ repair ability that CS/CNT/HA > CS/CNT > CS > control. Under the treatment of NIR irradiation, the epidermis thickness in CS + NIR, CS/CNT + NIR, CS/CNT/HA + NIR and Control + NIR groups were 141, 158, 230 and 100 μm, respectively. It enhanced significantly in CS/CNT + NIR and CS/CNT/HA + NIR groups when compared with the groups without NIR irradiation. Granulation tissues (composed of extracellular matrix, fibroblasts and many growth factors) played a vital role in wound healing, including absorbing necrotic tissue and filling cutaneous defects [64]. Therefore, granulation tissue thickness during wound repair is also a crucial indicator to evaluate the healing progress. In Fig. 10c, the granulation tissue thickness in CS, CS/CNT, CS/CNT/HA and control groups without NIR irradiation were 586, 649, 738 and 648 μm, respectively. In the groups of NIR irradiation, the corresponding thickness of granulation tissue was increased to 655, 668, 825 and 649 μm. It was worth noting that all the CS-based scaffolds had promoted the formation of granulation tissue and epidermis significantly when compared with the control. Besides, NIR treatment also enhanced the thickness of granulation tissue and newly formed epidermal hyperplasia obviously, especially in CS/CNT/HA group.

**Figure 11.**
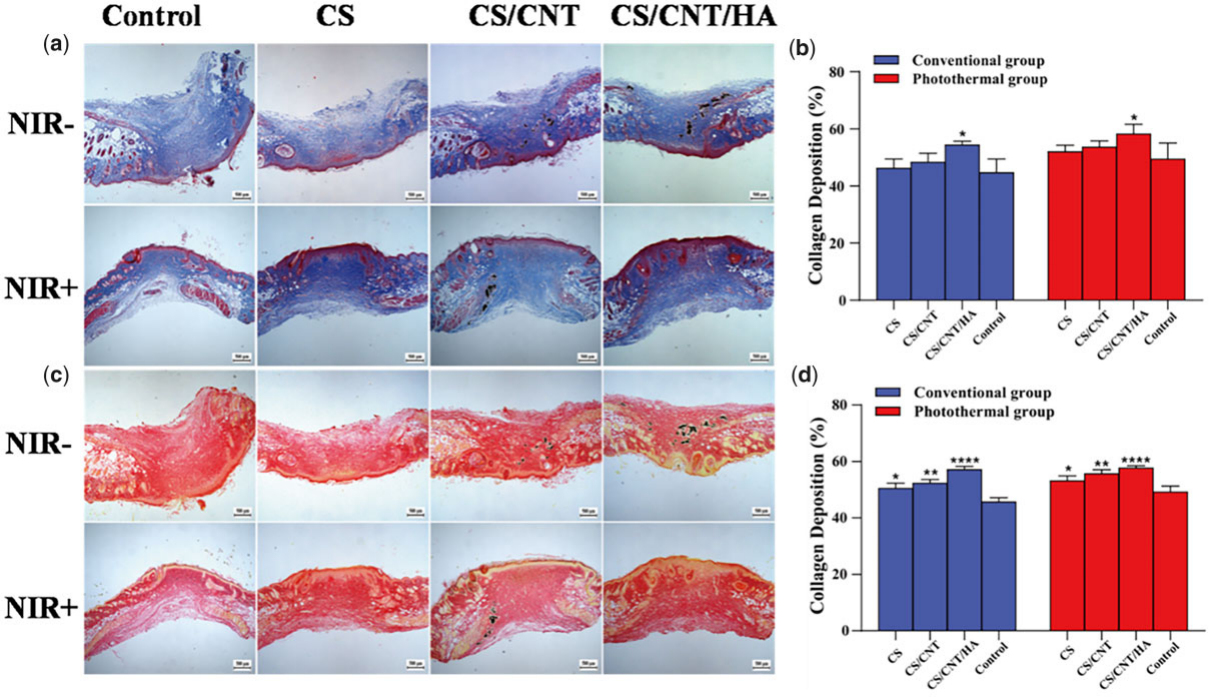
Histological analysis of CS, CS/CNT and CS/CNT/HA scaffolds in full-thickness skin wound healing using Masson trichrome staining (**a**) and Sirius red staining (**c**). The quantitative analysis of collagen deposition (**b** and **d**) in different groups according to the images of Masson trichrome staining and Sirius red staining (*difference of scaffolds compared with control, *P < 0.05, **P < 0.01 and ****P < 0.0001).

Collagen is the main component of the skin, which can provide elasticity and toughness to the skin and restore the structural integrity of the damaged tissues in wound healing [65]. The collagen deposition of the tissues was determined by Masson Trichrome and Sirius red staining. The proportion of collagen deposition in the wound area was measured by Image-Pro Plus 6.0 which was shown in Fig. 11. Masson Trichrome staining showed that the percentage of collagen deposition in the wound site without NIR irradiation were 46%, 48% and 54% for CS, CS/CNT and CS/CNT/HA, respectively, and the control group was 44%. After NIR irradiation, the collagen deposition areas of the three scaffolds were 52%, 53% and 58%, compared to only 49% in the control group. Sirius red staining results exhibited that the collagen deposition in three scaffolds without NIR irradiation was 50%, 52% and 57%, while the control group was only 45%. After being irradiated by NIR, the percentage of collagen in the wound area was only 49% in the control group, but that in the three scaffolds groups were up to 53%, 55% and 57%. It is obvious that the proportion of collagen deposition in the wound area in all three scaffolds was much larger than that in the control group either with NIR irradiation or not. Compared with the conventional group, the collagen deposition in the photothermal group was higher than that of the corresponding group. These results indicated that CS-based scaffolds could significantly accelerate the process of wound healing and promoted collagen deposition, especially the CS/CNT/HA scaffolds. Furthermore, the photothermal conditions also contribute to the formation of epithelial tissue, which was consistent with other reports [29]. In general, CS/CNT/HA scaffold is superior to other materials mainly ascribed to the excellent biological activity of n-HA released from the scaffold. The n-HA particles in CS/CNT/HA scaffolds showed a soft agglomerative state on a micro-scale and revert to nano-size after being degraded *in vivo*. As being reported by others, nano-scale HA particles can enter cells and regulate the cell behavior and function which participate the tissue repair, and finally accelerate wound regeneration [66–68].

## Conclusions

In this article, the CS-based porous scaffolds were prepared by freeze-drying. The microstructures, chemical properties, swelling properties, mechanical strength and antibacterial properties of the scaffolds were characterized. The results showed that the introduction of CNTs could produce a rapid thermal effect and superior antibacterial properties and could greatly improve the mechanical properties of CS scaffolds. Cell experiments indicated that all three scaffolds had appropriate biocompatibility and blood compatibility. Moreover, all three CS-based scaffolds showed a promotion on full-thickness skin repair as a wound dressing when compared to control. Importantly, the introduction of CNTs which have a photothermal response enhanced the repair effect, and the introduction of n-HA strengthened it further, indicated by the rapid regeneration of epithelial tissue and the deposition of collagen tissue. In particular, CS/CNTs/HA scaffold exhibited an optimal repair effect should be attributed to the synergistic effect of photothermal-responsive CNTs and bioactive n-HA.

## Ethical approval

In this study, all methods were carried out in accordance with relevant guidelines and regulations. All animal experiments were approved by the Institutional Animal Care and Use Committee of Northwestern Polytechnical University. All procedures on animals were in compliance with the animal protection agreements and regulations.
